# A conveyor belt experimental setup to study the internal dynamics of granular avalanches

**DOI:** 10.1007/s00348-021-03299-0

**Published:** 2021-09-25

**Authors:** Tomás Trewhela, Christophe Ancey

**Affiliations:** Laboratory of Environmental Hydraulics, École Polytechnique Fédérale de Lausanne, 1015 Écublens, Switzerland

## Abstract

**Abstract:**

This paper shows how a conveyor belt setup can be used to study the dynamics of stationary granular flows. To visualise the flow within the granular bulk and, in particular, determine its composition and the velocity field, we used the refractive index matching (RIM) technique combined with particle tracking velocimetry and coarse-graining algorithms. Implementing RIM posed varied technical, design and construction difficulties. To test the experimental setup and go beyond a mere proof of concept, we carried out granular flow experiments involving monodisperse and bidisperse borosilicate glass beads. These flows resulted in stationary avalanches with distinct regions whose structures were classified as: (i) a convective-bulged front, (ii) a compact-layered tail and, between them, (iii) a breaking size-segregation wave structure. We found that the bulk strain rate, represented by its tensor invariants, varied significantly between the identified flow structures, and their values supported the observed avalanche characteristics. The flow velocity fields’ interpolated profiles adjusted well to a Bagnold-like profile, although a considerable basal velocity slip was measured. We calculated a segregation flux using recent developments in particle-size segregation theory. Along with vertical velocity changes and high expansion rates, segregation fluxes were markedly higher at the avalanche’s leading edge, suggesting a connection between flow rheology and grain segregation. The experimental conveyor belt’s results showed the potential for further theoretical developments in rheology and segregation-coupled models.

**Graphic Abstract:**

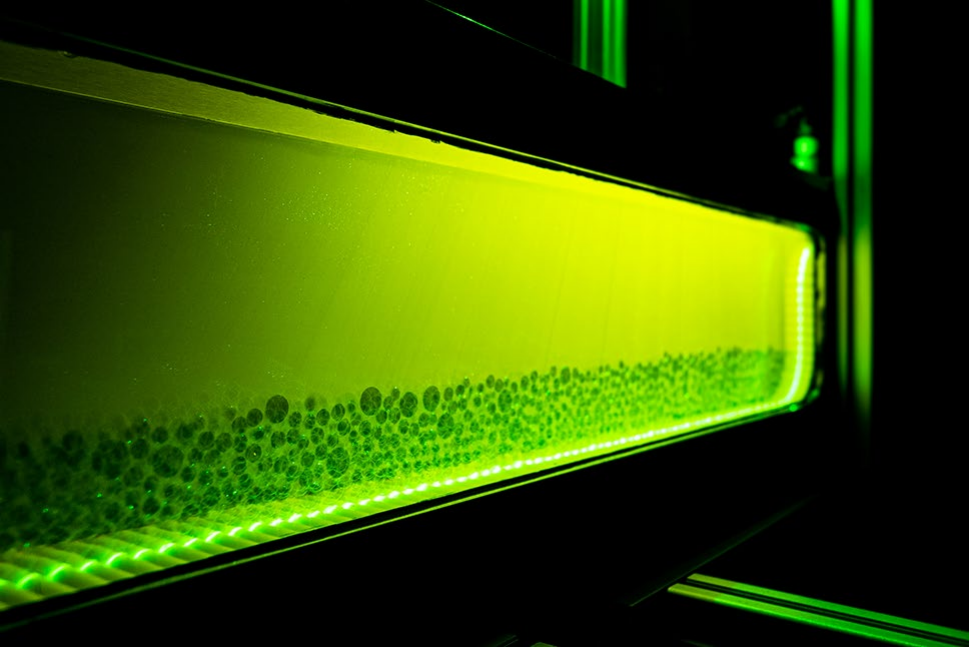

## Introduction

Granular flows are often studied using steady-state experiments in long flumes with constant feed rates (e.g. MiDi 2004; Delannay et al. 2017). Dam-break experiments, in which a finite volume of granular material is released down a slope, have also been performed (e.g. Savage and Hutter 1989; Pouliquen 1999; Gray and Ancey 2009; Johnson et al. 2012; Saingier et al. 2016), but less frequently given the difficulty of capturing internal front dynamics. A convenient way to remove this difficulty is to create a steady granular avalanche on a conveyor belt (Davies 1988, 1990). Conveyor belts have a mobile, rough bottom that drags materials from one place to another, and they are commonly used in industry (e.g. Dhillon 2008; Pane et al. 2019). If a granular material flows under the effect of gravity while subject to a controlled drag that counterbalances the gravitational effect, the resulting flow should reach a steady state. Looking at this particular situation is then equivalent to moving a camera with the flow front in a dam-break experiment.

The internal dynamics of granular avalanches affect their propagation. For instance, their runout distance depends crucially on grain friction and composition (Linares-Guerrero et al. 2007; Roche et al. 2008; Iverson et al. 2010; Mangeney et al. 2010; Kokelaar et al. 2014), whereas self-generated internal structures such as levees (Deboeuf et al. 2006; Mangeney et al. 2007; Rocha et al. 2019) and breaking size-segregation waves (Gray and Ancey 2009; Gajjar et al. 2016; van der Vaart et al. 2018) or reverse segregation (Thomas and D’Ortona 2018) regulate the spread of the flow. Polydisperse granular materials are prone to separate themselves by particle size, in a process called particle-size segregation (Gray 2018). In gravity-driven shallow granular avalanches, large particles are often encountered in the flow’s leading edge and near the free surface, whereas small grains are more likely to be found in the tail and along the bottom (Gray and Thornton, 2005; Gray and Ancey 2009; Johnson et al. 2012; Gray 2018).

For segregating granular flows, Thornton and Gray (2008) worked out an analytical solution for a inversely graded inflow (i.e. a flow where the large particles are on top of small particles). They investigated a boundary initial value problem, in which the particles were initially separated and formed a step (see their Fig. 1). When the particles were set in motion, the flow was initially stable (from segregating particles) since all particles were separated out into two segregated layers, with the large particles above the small ones. However, this configuration became rapidly unstable as the shearing caused the upper layers to move faster than the lower layers: the sharp interface between both grain classes steepened progressively. This eventually resulted in a breaking wave when the overhanging crest induced an unstable stratification. To investigate breaking size-segregation waves, Thornton and Gray (2008) used the simple continuum model for segregation in bimodal granular flows, derived by Gray and Thornton, (2005). In spite of recent efforts to describe these waves (e.g. Johnson et al. 2012; van der Vaart et al. 2018), their impact on the flow’s rheology or propagation remains poorly understood. To appropriately investigate the connection between flow and segregation, it is thus important to create the experimental conditions that make it possible to observe flows with well-defined fronts and subject to inversely graded particle arrangements.

A better understanding of the link between the flow dynamics and internal structure of granular flows is central to modelling them. Experimentally, this understanding can be achieved by visualising and measuring flow characteristics within the granular bulk. Among the laboratory-scale methods used for studying granular flows, techniques based on refractive index matching (RIM) have been used increasingly in recent years (Budwig 1994; Wiederseiner et al. 2011; Dijksman et al. 2012; Sanvitale and Bowman 2016; Poelma 2020). Applying RIM to granular avalanches is fraught with difficulties, however. When the setup is not immersed, light-scattering (Byron and Variano 2013), free-surface effects and bubbles (Cui and Adrian 1997) are problematic. Immersing the whole setup in an index-matched fluid usually solves these problems, but this solution has the disadvantage of requiring large volumes of fluids in long inclined flumes (van der Vaart et al. 2018). Another difficulty common to RIM techniques is the limited number of fluid-grain combinations that come close to real-world magnitudes of fluid viscosity and density ratios (Wiederseiner et al. 2011; Dijksman et al. 2012).

Since Davies (1988)’s pioneering experiments, conveyor belts have been used increasingly to study granular flows (Perng et al. 2006; Martínez 2008; Marks et al. 2017; van der Vaart et al. 2018). The articles by Marks et al. (2017) and van der Vaart et al. (2018) deserve special mention because of their focus on particle-size segregation. Marks et al. (2017) studied size segregation in stationary avalanches using a two-dimensional conveyor belt. One drawback of this configuration is that it leads to flow features that are not observed in three-dimensional configurations, i.e. significant sidewall effects, convection cells and reduced percolation of the finest grains (Thomas and Vriend 2019; Trewhela et al. 2021b). van der Vaart et al. (2018) found mobility-feedback dynamics similar to those of (Marks et al. 2017), but using a three-dimensional conveyor belt.

This paper presents an experimental three-dimensional setup based on a conveyor belt and RIM techniques that we used to study stationary granular avalanches. The setup was an enhanced version of the prototype built by van der Vaart et al. (2018). During their preliminary experiments, van der Vaart et al. (2018) identified various technical difficulties requiring solutions. This work describes these problems and how we solved them. We then conducted experiments using sets of monodisperse and bidisperse granular media to investigate the dynamics of stationary granular avalanches. We provide a qualitative and quantitative picture of these avalanches by showing their bulk compositions, velocity profiles, strain-rate tensor invariants and segregation fluxes.

## Theoretical framework

### Granular flow equations

**Fig. 1:**
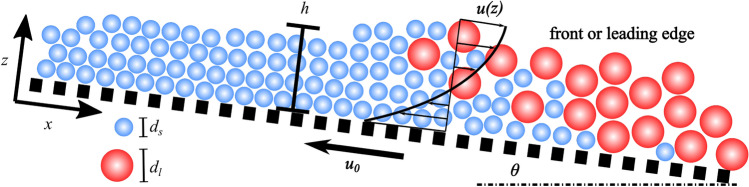
Sketch for the theoretical framework of the conveyor belt flow configuration. A stationary avalanche flows over an inclined moving plane forming a front or leading edge. For bidisperse avalanches, this front usually concentrates large particles of size $d_{l}$ (blue circles) that recirculate within the front’s bulk due to segregation

We consider a conveyor belt inclined at $\theta$ to the horizontal, creating a stationary avalanche that travels at the same mean speed $u_{0}=u_{b}$ of the moving belt (see Fig. 1). We assume that in this flow configuration, the granular avalanche can be decomposed into a steady uniform layer and a leading edge. The momentum balance equation for the uniform layer is

1 $$\begin{aligned} \frac{\mathrm {d}\tau _{xz}}{\mathrm {d}z}&=-\varrho g \mathrm {sin}\;\theta , \end{aligned}$$

2 $$\begin{aligned} \frac{\mathrm {d}\sigma _{z}}{\mathrm {d}z}&=\varrho g \mathrm {cos}\;\theta , \end{aligned}$$

where $\tau _{xz}$ is the shear stress, $\sigma _{z}$ is the normal stress in the *z*-direction, and $\varrho =\varrho _{*}-\varrho ^{f}_{*}$ denotes the reduced bulk density defined by the fluid $\varrho ^{f}_{*}$ and particles $\varrho _{*}$ intrinsic densities (Table 1). The *x*-direction is aligned with the bottom and the *z* direction is normal to the bottom. We assumed a stress-free condition at the free surface $z=h$. Integrating Eqs. (1) and (2) leads to the well-known shear and normal stress distributions that hold independently of the bulk’s constitutive equations:

3 $$\begin{aligned} \tau _{xz}(z)&=\varrho g \mathrm {sin}\;\theta \;(h-z), \end{aligned}$$

4 $$\begin{aligned} \sigma _{z}(z)&=-\varrho g \mathrm {cos}\;\theta \;(h-z). \end{aligned}$$

For granular flows in a frictional-collisional regime, bulk stresses are generated by collisional and frictional contacts between particles. Ancey and Evesque (2000) argued that for this regime to hold, the constitutive equation should depend on a dimensionless number that reflects the balance between these antagonistic contact forces

5 $$\begin{aligned} I=\frac{{\dot{\gamma }}d}{\sqrt{\sigma _{z}/\varrho }}, \end{aligned}$$

where ${\dot{\gamma }}=|\mathrm {d}u/\mathrm {d}z|$ is the shear rate and *d* is the particle diameter. This dimensionless number was subsequently renamed inertial number (MiDi 2004). By compiling experimental data, Jop et al. (2006) deduced a generalised Coulomb relationship called the $\mu (I)$ rheology: $\tau _{xz}=\mu (I)\sigma _z$, where $\mu$ denotes the bulk friction coefficient. Under steady uniform flow conditions, Eqs. (3) and (4) also impose that $\tau _{xz}/\sigma _z=\tan \theta$, and thus $\mu (I)=\tan \theta$. Inversing this condition leads to the following expression of the inertial number $I$ for a given slope $\theta$:

6 $$\begin{aligned} I=I_{0}\frac{\mathrm {tan}\;\theta -\mu _{1}}{\mu _{2}-\mathrm {tan}\;\theta }, \end{aligned}$$

where $I_{0}$, $\mu _{1}$ and $\mu _{2}$ are empirical parameters. This equation implies that a steady uniform flow can be achieved with a limited range of inclinations: $\mu _{1}<\mathrm {tan}\;\theta <\mu _{2}$ (Jop et al. 2006). This expression along with Eq. 5 and the basal velocity condition $u_{0}=u_{b}$ (where $u_{b}$ denotes the belt velocity) yields a Bagnold-like velocity profile

7 $$\begin{aligned} u(z)=-u_{b}+\frac{2I\sqrt{g\mathrm {cos}\;\theta h^{3}}}{3d}\left[ 1-\left( 1-\frac{z}{h}\right) ^{3/2}\right] . \end{aligned}$$

This velocity profile is characteristic of steady uniform granular flows of monodisperse grains (Silbert et al. 2001; Mitarai and Nakanishi 2005). When granular flows involve polydisperse materials, they may exhibit a different velocity profile due to segregation-induced grain rearrangement (Tripathi and Khakhar 2011).

### Size segregation equations

The internal composition of the stationary granular avalanches can be studied in terms of particle movement and size segregation by using a continuum approach. For a bidisperse granular mixture of grains of different diameters, the volumetric concentrations of the particle species satisfy

8 $$\begin{aligned} \sum _{\nu } \phi ^{\nu } = 1, \end{aligned}$$

where $\phi ^{\nu }$ is the partial volume fraction for each grain species $\nu =\lbrace s,l\rbrace$, which is characterised by a diameter $d_{\nu }$. The bidisperse size segregation equation for the $\nu$ species is then given by (Gray 2018):

9 $$\begin{aligned} \frac{\partial \phi ^{\nu }}{\partial t}+ \nabla \cdot (\phi ^{\nu }{\varvec{u}})+\nabla \cdot {\varvec{F}}^{\nu }=\nabla \cdot ({{\mathcal {D}}_{sl} \nabla \phi ^{\nu }}), \end{aligned}$$

where ${\varvec{u}}$ is the bulk velocity and ${\varvec{F}}^{s}=f_{sl}\phi ^{s}\phi ^{l} {\varvec{g}}/|{\varvec{g}}|,\; {\varvec{F}}^{l}=-f_{sl}\phi ^{s}\phi ^{l} {\varvec{g}}/|{\varvec{g}}|$ are the segregation fluxes, which are oriented with the direction of gravity and satisfy:

10 $$\begin{aligned} \sum _{\nu } {\varvec{F}}^{\nu } = \varvec{1}. \end{aligned}$$

In a stationary regime, and in the absence of diffusion, we can simplify Eq. (9) and obtain

11 $$\begin{aligned} {\varvec{u}}^{l}-{\varvec{u}}&= -f_{sl}(\phi ^{s})\phi ^{l} {\varvec{g}}/|{\varvec{g}}|, \end{aligned}$$

12 $$\begin{aligned} {\varvec{u}}^{s}-{\varvec{u}}&= f_{sl}(\phi ^{s})\phi ^{s}{\varvec{g}}/|{\varvec{g}}|, \end{aligned}$$

where $f_{sl}$ corresponds to the theoretical segregation flux function for large and small particles. This function has been proposed to be cubic (Bridgwater et al. 1985), quadratic (Dolgunin and Ukolov 1995), asymmetric (Gajjar and Gray 2014; van der Vaart et al. 2015) and highly nonlinear (Trewhela et al. 2021a). The asymmetric nature of the size segregation phenomenon is highly significant: small particles segregate faster than their large counterparts. This asymmetry has been observed in numerical simulations and laboratory experiments (Gajjar and Gray 2014; van der Vaart et al. 2015; Jones et al. 2018; Trewhela et al. 2021a).

The experimental results presented in this paper used a coarse-graining technique to infer the continuous distributions of the bulk density $\varrho$, partial concentrations $\phi ^{\nu }$ and velocity fields ${\varvec{u}}$ from particle positions ${\varvec{r}}_{i}$ and velocities ${\varvec{u}}_{i}$ determined experimentally (e.g. Goldhirsch 2010; Tunuguntla et al. 2016) (see Sect. 3.3 for further information). The velocity fields for each species ${\varvec{u}}^{\nu }$ were also computed. Based on these computations and following the recent work of Trewhela et al. (2021a), we defined $f_{sl}$ as

13 $$\begin{aligned} f_{sl}={\mathcal {B}}\frac{\varrho _{*}g{\dot{\gamma }} d^{2}}{{\mathcal {C}} \varrho _{*}gd+p}{\mathcal {F}}(R,\phi ^{s}), \end{aligned}$$

where ${\mathcal {B}}=$ 0.3744 and ${\mathcal {C}}=$ 0.2712 are two constants determined from segregation experiments in a three-dimensional shear box. ${\mathcal {F}}$ is a function that depends mostly on the size ratio $R=d_{l}/d_{s}$, for intermediate values of $\phi ^{s}$, and will be considered to be equal to $R-1$. ${\dot{\gamma }}$ is the shear rate, ${\text{d}}$ is the concentration-averaged diameter

14 $$\begin{aligned} d=d_{s}\phi ^{s}+d_{l}\phi ^{l}, \end{aligned}$$

and $p=\varrho _{*}g\Phi (h-z)\mathrm {cos}\;\theta$ is the pressure, considered to be lithostatic for a flow of height ${\text{h}}$. An expression for ${\text{d}}$ as a function of $\phi ^{s}$ was then derived:

15 $$\begin{aligned} d=Rd_{s}\left[ 1-\left( 1-\frac{1}{R}\right) \phi ^{s}\right] =Rd_{s}d_{\phi }. \end{aligned}$$

By inserting the latter into Eq. (13), we determined a simplified segregation flux function

16 $$\begin{aligned} f_{sl}={\mathcal {B}}\frac{{\dot{\gamma }} (Rd_{s}d_{\phi })^{2}}{{\mathcal {C}} Rd_{s}d_{\phi }+\Phi (h-z) \mathrm {cos}\;\theta }(R-1). \end{aligned}$$

The shear rate calculation, which involved the strain-rate tensor invariants, is detailed in the next subsection.

From the definition of the concentration-averaged diameter (Eq. 14) relative to the particles’ diameters, it is possible to obtain the expression

17 $$\begin{aligned} \frac{d-d_{s}}{d_{s}-d_{l}}=\frac{d_{s}\phi ^{s}+d_{l}(\phi ^{l}-1)}{d_{s}-d_{l}}=\phi ^{s}. \end{aligned}$$

The expression proposed by Trewhela et al. (2021a) (Eq. 13) was developed from experiments (in a three-dimensional shear box) to account for the asymmetric nature of size segregation. The expression’s segregation timescale was set by the shear rate and depended on the size difference ${R}$ and pressure distribution *p*. Their expression has also been used in numerical simulations (Barker et al. 2021) and validated for two-dimensional experiments (Trewhela et al. 2021b). Moreover, in our conveyor belt experiments we used precisely the same fluid and beads as those used by Trewhela et al. (2021a), with ${R}$ values within the range given in their work. Based on the comparisons made by Trewhela et al. (2021a) with the previous work of van der Vaart et al. (2015) and Thornton et al. (2012), the values of ${\mathcal {B}}$ and ${\mathcal {C}}$ were found pertinent for their use in this work.

### Strain-rate tensor invariants

Based on the velocity field ${\varvec{u}}$, the strain-rate tensor is defined as

18 $$\begin{aligned} {\varvec{D}}= \frac{1}{2}[\nabla {\varvec{u}}+(\nabla {\varvec{u}})^{T}], \end{aligned}$$

where $T$ denotes the transpose. The strain-rate tensor’s first invariant, also called the expansion rate, can be calculated as

19 $$\begin{aligned} I_{{\varvec{D}}}= \text {tr}({\varvec{D}})=\varvec{\nabla \cdot u}. \end{aligned}$$

A tensor decomposition determines the deviatoric strain-rate tensor ${\varvec{S}}=-\frac{1}{2}I_{{\varvec{D}}}{\varvec{1}}+{\varvec{D}}$, which is useful for calculating the strain-rate tensor’s second invariant:

20 $$\begin{aligned} II_{{\varvec{D}}}=\left[ \frac{1}{2}\text {tr}({\varvec{S}}^{2})\right] ^{1/2}, \end{aligned}$$

where ${\dot{\gamma }}=2II_{{\varvec{D}}}$. Throughout this paper, mentions of or discussions on the shear rate refer to the strain-rate tensor’s second invariant.

## Materials and techniques

### Refractive index matching

Most granular materials are opaque, and in most experimental facilities, this property restricts any inspection of them to their boundaries. Even when grains are transparent, refractive index differences between the interstitial medium and the grain material impede observation within the granular bulk. To overcome such natural restrictions, it is desirable to match the refractive indices of the granular material and the interstitial fluid. Furthermore, using laser-induced fluorescence, light can be shone through the bulk, making the grains appear as dark shapes. This technique is possible in a laboratory environment if the fluid temperature is stable (as its refractive index retains a known value). This is called refractive index matching (RIM) and has been used not only to study granular flows but also other fluids (Budwig 1994; Li et al. 2005; Wiederseiner et al. 2011; Dijksman et al. 2012; Bai and Katz 2014; Clément et al. 2018; Rousseau and Ancey 2020).

The present study used a RIM mixture composed of borosilicate glass beads immersed in a fluid solution of ethanol and benzyl alcohol. Their refractive indices $n_{r}$, densities $\varrho _{*}$ and suppliers are detailed in Table 1. Two additional RIM mixtures were considered for this study but rejected due to their interstitial-fluid properties. The first alternative was a combination of Triton X-100 fluid and poly (methyl methacrylate) particles (PMMA) (for details on this combination, see Dijksman and van Hecke 2010; Wiederseiner et al. 2011; Dijksman et al. 2012). This was rejected due to the fluid’s high viscosity ($\eta =$ 270 cP) and the low density difference between the particles and the fluid, ${\hat{\varrho }}=(\varrho _{*}-\varrho ^{f}_{*})/\varrho^{f} _{*}=0.102$. The second was a combination of an aqueous sodium iodide solution (for studies using this combination, see Narrow et al. 2000; Bai and Katz 2014; Clément et al. 2018) and borosilicate glass beads, but the interstitial fluid was too dense and the beads showed positive buoyancy. Finally, we retained borosilicate glass for the particles and a mixture of ethanol and benzyl alcohol for the interstitial fluid–a combination recently used by van der Vaart et al. (2015) and Rousseau and Ancey (2020). The liquid mixture’s viscosity was close to that of water: $\eta \approx$ 3 cP. The density contrast between the borosilicate and the fluid mixture was negative and sufficient to replicate the physics of wet granular flows ${\hat{\varrho }}\approx 1.34$.

**Table 1:** Refractive indices $n_{r}$, intrinsic densities $\varrho _{*}$ and suppliers of the materials used for our RIM experiments

Material	$n_{r}$	$\varrho _{*}$ (g $\hbox {cm}^{-3}$)	Supplier
Borosilicate glass	1.4726	2.23	Schäfer glass
Benzyl alcohol	1.5396	1.044	Acros organics
Ethanol	1.3656	0.789	Fisher scientific

The beads’ refractive index $n_{r}=1.4726$ (Table 1) was initially matched using 35 parts ethanol and 65 parts benzyl alcohol by weight (Chen et al. 2012). We then tuned the mixture’s refractive index by adding small volumes of either ethanol or benzyl alcohol until the precise index was obtained. The $n_{r}$ was constantly measured during this adjustment stage using a Atago RX 5000 $\alpha$ refractometer in a 20 $^{\circ }\hbox {C}$ temperature-controlled environment. It was impossible to reduce uncertainties below $\pm 2\times 10^{-4}$ because of the large volume of fluid required for our experiments (about 40 L). Although this large volume was mixed using a motorised mixer, it was difficult to obtain a perfectly homogeneous mixture. Furthermore, the mixing process caused some ethanol to evaporate, leading to a slight mismatch in the fluid’s refractive index and the formation of bubbles that enhanced the evaporated ethanol’s carriage to the surface.

### Image acquisition

The RIM technique is often combined with laser-induced fluorescence (Sanvitale and Bowman 2012, 2016). When used alone, RIM produces a transparent medium, and it is impossible to distinguish between the fluid and solid phases. By mixing a fluorescent dye into the fluid and exciting it using a laser sheet, the solid phase can be made visible to a given light wavelength. For our experiments, we mixed our RIM fluid with a small amount of Rhodamine 6G (Acros Organics) and used a 4W Viasho laser with a $\lambda =$ 532 nm wavelength (green) to produce the laser sheet. When filmed, particles then appeared as black circles. We used a Basler A403k camera mounted with a 28-mm Nikon lens, operated at a fixed rate of 40 frames per second for all experiments; image resolution was of $2352 \times 600$ pixels.

### Particle tracking and coarse-graining

Image sequences were analysed using circle identification and particle tracking algorithms. The imfindcircles algorithm included in Matlab was used to distinguish the various-sized circles. Particles’ positions were determined over image sequences and then correlated using the tracking algorithm developed by Crocker and Grier (1996) to obtain particle trajectories ${\varvec{r}}_{i}(x,z,t)$ over time, and particle velocities ${\varvec{u}}_{i}(x,z,t)$.

One recurring difficulty in studying granular flows involves transforming discrete information into continuous profiles or fields–an indispensable step for comparing experimental data and the predictions from continuum models. To overcome this problem, coarse-graining techniques have been developed to convolute discrete experimental and numerical results into continuum fields (e.g. Weinhart et al. 2012; van der Vaart et al. 2015; Tunuguntla et al. 2016). The advantages of coarse graining are numerous (Goldhirsch 2010), for example: (i) it is possible to obtain continuous, smooth, differentiable fields and profiles, which is a particularly helpful feature close to the boundaries; and (ii) the fields derived satisfy both mass and momentum conservation equations (provided that the coarse-graining functions are differentiable and integrable). In our experimental analysis, we used a fourth-degree Lucy polynomial (Lucy 1977) previously used in the post-analysis of discrete particle simulations (Tunuguntla et al. 2016). For this study, the width function or coarse-graining scale, i.e. ${w}$ in Tunuguntla et al. (2016), was defined equal to ${\bar{d}}=\Phi ^{s}d_{s}+\Phi ^{l}d_{l}$. Coarse-graining is instrumental to providing a point of comparison between discrete experimental measurements and continuum theories, but it has a drawback. By using the technique, we blurred grain-scale information and smoothed grain-grain interactions or dramatic changes in velocities or concentrations. As we were not interested in a grain-scale resolution, we did not explore alternative techniques such as the one proposed by Ni and Capart (2015) which makes it possible to infer information at the grain scale.

## The experimental conveyor belt setup

**Fig. 2:**
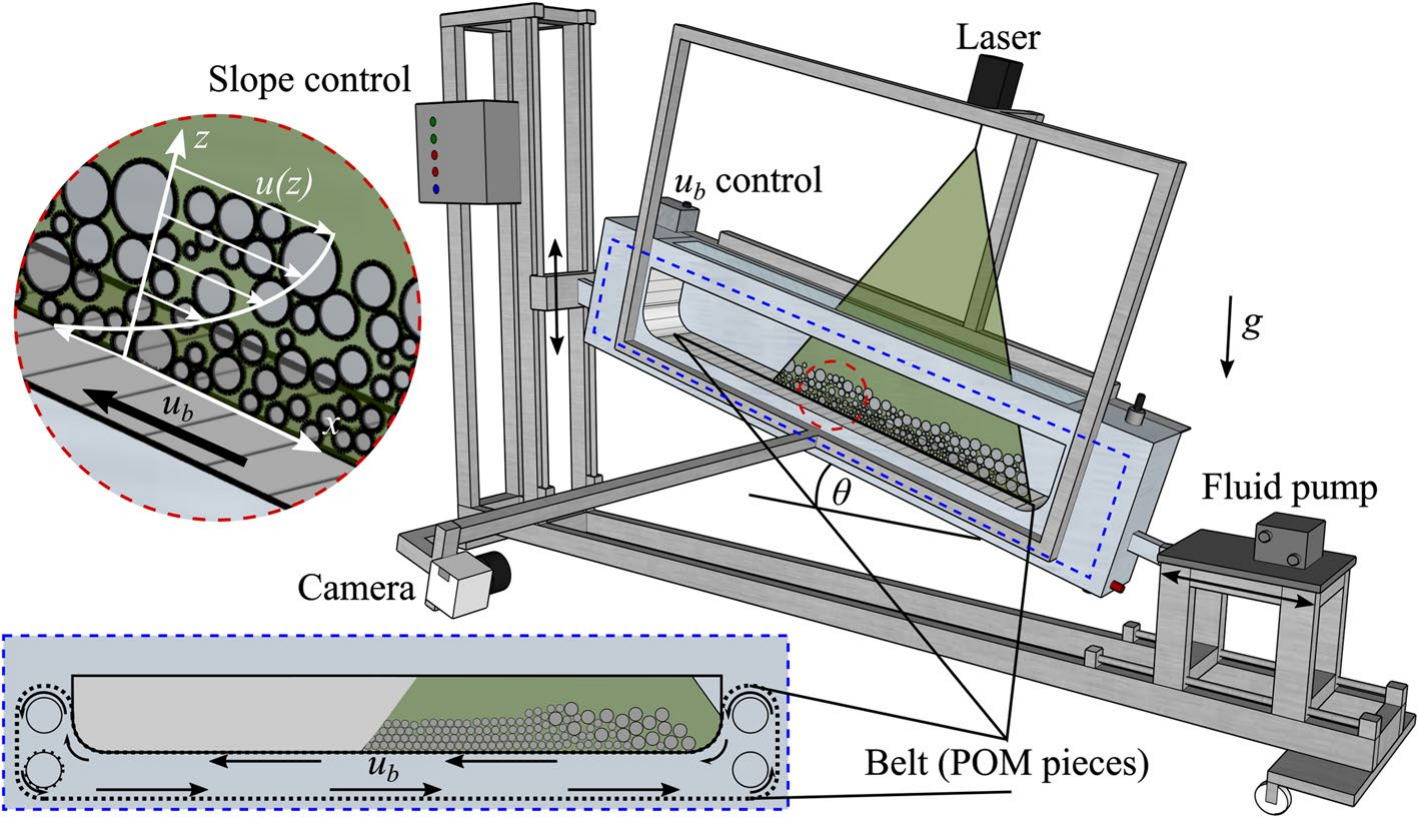
Diagram of the conveyor belt setup. The enclosed aluminium flume (centre) is closed off by a rectangular windowed lid that allows the passage of the laser sheet from above. A rough belt made from 300 independent pieces of POM circulates around the flume, guided via grooves in its front and back aluminium walls. The slope control (left) sets the vertical position of the flume’s uphill end, with its downhill end attached to a sliding chariot where the fluid pump rests (right). Conveyor belt velocity $u_{b}$ is controlled electronically (centre). The laser sheet was aligned longitudinally to the flume and passed through the middle of the bulk in the transverse direction ($y=$ 5 cm). An example of the visualised longitudinal section of the flow is shown within the blue dashed frame (bottom left corner). Images were acquired using a Basler A403k camera placed in the front of the flume and equipped with a Nikon 28-mm lens and a 532-nm filter. A movie showing the operation of the conveyor belt is available online

We designed and built a conveyor belt flume to study stationary granular flows (see Fig. 2). The setup included an aluminium flume 141 cm long, 14 cm wide and 42 cm high. A sealed rectangular panel with a glass window was placed on top allowing the laser attached to the setup to create a laser sheet perpendicular to it. Two grooves carved in the flume’s front and back aluminium panels guided the longitudinal movement of the rough conveyor belt made up of 300 independent pieces and around four transversal aluminium rollers, with one pair located at each end of the aluminium flume. Each roller pair was arranged vertically to create walls that confined the granular material to the conveyed volume over the belt’s moving parts. The conveyed volume was 104 cm long, 10 cm wide, and 15 cm high. The aluminium flume had a glass window in the sidewall, parallel to the flow and compatible with image acquisition and the visualisation of the entire conveyed volume. A mixer was located behind the pair of rollers at each end of the flume’s aluminium structure to homogenise fluid. Various valves, beneath and above the setup, helped the processes of filling and emptying it.

An analogue electro-mechanical system set the flume’s slope by vertically adjusting the flume’s uphill end. The flume’s downhill end (fixed to a mobile cart) moved simultaneously in the horizontal direction. The slope could be adjusted to a wide range of values. Gentle or very steep slopes were inadvisable and impractical because particles might overflow the upper rollers and create mechanical issues.

Each independent piece of the rough belt was a half-cylinder of polyoxymethylene (POM) screwed to an aluminium band. Roughness could be changed easily by replacing the POM half cylinders. For our experiments, we used a uniform roughness given by half cylinders of 4 mm in radius. Pieces were inserted into the grooves, next to each other, and were kept in place by the compression that the pieces applied against each other and were restrained vertically by the grooves.

The conveyor belt was operated using a motor located behind the setup. The motor rotated the bottom uphill roller, whose geared wheel pushed the POM pieces along between the grooves in the sidepanels. Half of the pieces had two bolts beneath them so that the geared wheel could push against them and, move the belt. Pieces were alternately bolted and non-bolted to avoid breaking the geared wheel, the roller or the POM pieces. Motor speed was controlled using a dimmer switch that could apply a continuous range of belt velocities $u_{b}$. The analogue controller did not give the $u_{b}$ value directly, and it had to be measured using a sensor that counted the motor axis revolutions as a function of time. These revolutions were then easily translated to a precise $u_{b}$ value using the radius of the geared wheel.

The friction coefficient of the belt could not be determined as a single constant value. As a result of their fabrication, the POM pieces obtained a particular roughness which could not be determined precisely so values from the literature were considered. According to Vaziri et al. (1988), POM has a low speed frictional coefficient $\mu _{ls}$ of 0.16 and 0.24 for like-on-like essays using cylinders and flat surfaces, respectively. Unfortunately, they did not carry essays of a POM cylinder sliding against glass surfaces or beads. The belt’s relative roughness to the particles’ diameters was also relevant for friction. In our experiments, the ratio between the pieces’ and the particles’ diameters were 1.33, 1 and 0.57 for $d_{s,l}=$ 6, 8 and 14, respectively. In this sense, the belt roughness sought to recreate a rough lower boundary condition as if it was made by the same glass beads forming the bulk. This type of boundary condition is commonly used in numerical simulations (e.g. Tunuguntla et al. 2016; van der Vaart et al. 2018).

Sidewall effects are always present in this type of flumes (e.g. Jop et al. 2005). To avoid major influence from the side walls, We used the RIM technique to visualise the bulk from within and by doing so, we avoided major influence from the side walls. The laser sheet was placed in the middle of the flume’s transverse direction, i.e. at $y=$ 5 cm from each side of the flume (see Fig. 2).

For the reader interested on other setup-related issues, see § A.

## Experimental dataset

**Fig. 3:**
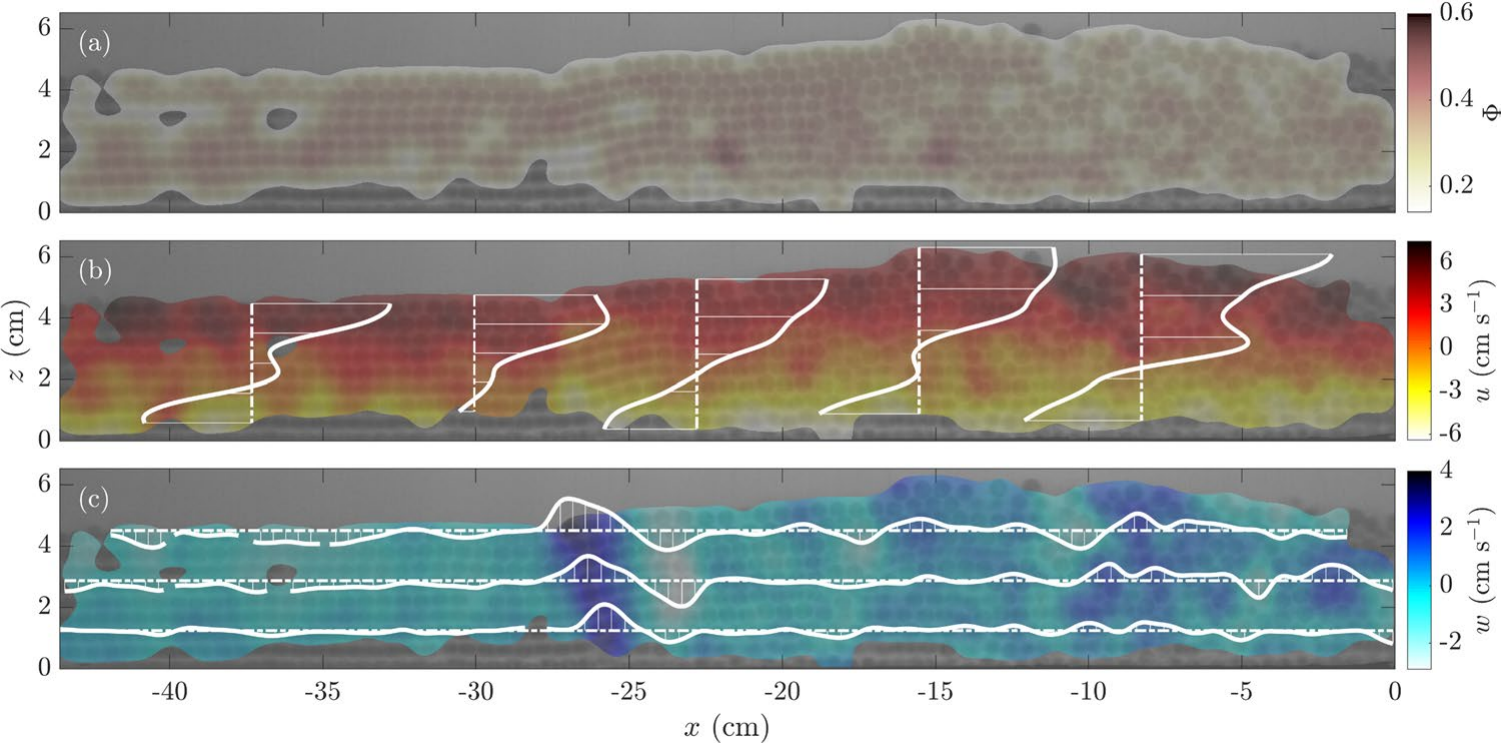
**a** Bulk’s solids volume fraction $\Phi$,** b** longitudinal velocity field ${u}$ and** c** vertical velocity field ${w}$ at $t=$ 229.25 s for Experiment 1 (see Table 2). Longitudinal distances were measured from the wall formed by the POM pieces passing around the flume’s downhill pair of rollers. Two differing flow sections can be distinguished from the images with a sharp transition at $x\approx$ − 25 cm: (i) for $x\lesssim$ − 25 cm, a well-arranged particle flow flowing in layers, and; (ii) for $x\gtrsim$ − 25 cm, a convective-bulged front where particles recirculate. The discontinuous lines correspond to** b** vertical or** c** horizontal profiles of the velocity field. Velocity profile values are plotted using continuous white lines. These values are only shown to illustrate relative fluctuations along the profiles and visualise their shape. A movie of Experiment 1 is available as supplementary material

**Table 2:** Parameters of the experimental dataset. $\Phi ^{s}$ is the overall small particle proportion of the bulk, the slope $\theta$ and the measured speed of the conveyor belt $u_{b}$

Experiment no.	$d_{s}$ (mm)	$d_{l}$ (mm)	$\Phi ^{s}$ (%)	$\theta$ ($^{\circ }$)	$u_{b}$ (cm $\hbox {s}^{-1}$)
1 (monodisperse)	6	–	100	15	8.16
2 (bidisperse)	6	14	90	15	7.74
3 (bidisperse)	6	14	80	15	7.76
4 (bidisperse)	6	14	70	15	8.24
5 (bidisperse)	6	14	60	15	7.62
6 (monodisperse)	8	–	100	15	7.94
7 (bidisperse)	8	14	90	15	7.69
8 (bidisperse)	8	14	80	15	8.09
9 (bidisperse)	8	14	70	15	7.82
10 (bidisperse)	8	14	60	15	8.16

The conveyor belt was used to study the internal dynamics of granular flows made of monodisperse or bidisperse media. Conveyor belt velocity and slope were adjusted so that stationary avalanches could be observed in the flume. Experiments were thus characterised by the slope $\theta$ and belt velocity $u_{b}$ (see Table 2). All the experiments presented here were carried out with the flume inclined at $\theta =$15°  to the horizontal.

Two experiments were initially carried out using monodisperse beads to determine a base state for later comparison with bidisperse experiments. The granular material used for these two runs was a 6-kg bulk of borosilicate beads of either $d_{s}=$ 6 or 8 mm diameter (see Experiments 1 and 6 in Table 2). The choice for the particles’ diameters in the bidisperse experiments ($d_{s}=\lbrace 6,8\rbrace$ and $d_{l}=14$) was based on previous studies where different segregation mechanics were observed for size ratio *R* values lower and higher than 2 (Trewhela et al. 2021a, b). We determined bulk concentrations and velocity fields as functions of time. Next, we time-averaged these fields to obtain general trends and to describe processes that instantaneous frames were unable to show.

The main body of experimental work consisted of eight stationary bidisperse granular avalanches. To include large particles in the bulk, we replaced part of the weight of small particles with large particles keeping the same total weight and only changed $\Phi ^{s}$. The overall general small particle concentration $\Phi ^{s}$, therefore, ranged from 90 to 60%, with the large particle concentration varying complementarily, i.e. $\Phi ^{l}=100-\Phi ^{s}$. Since both species had the same intrinsic material density $\varrho _{*}$, the overall bulk volume concentration remained the same in every run. In addition to bulk concentrations and velocity fields, local volume concentrations of small particles $\phi ^{s}$ and large particles $\phi ^{l}=1-\phi ^{s}$ were also determined from the images acquired using the coarse-graining technique (see Sect. 3.3) for experiments involving bidisperse media.

Before data acquisition, the experiments were run until a stationary and, when possible, uniform flow was achieved. For each experiment, we first prepared the granular bulk of small and large particles (when needed) in the desired proportions. The particles were then put inside the flume and mixed as it rested almost horizontally. This resting position prevented particles from moving and altering their initial arrangement. We mixed the bulk before each experiment to have a close-to-homogeneous and reproducible initial condition. The fluid pump (shown in Fig. 2) was used to fill the flume with the interstitial fluid, and once full, we inclined the flume and turned on the motor. Only after stationary flow condition was achieved, image acquisition could begin. The flow was considered stationary when the flow’s height profile and the avalanche front did not vary notably over several minutes. Image acquisition was performed for 5 minutes, and we took 12,000 frames per run (i.e. 40 frames per second). The flume was then returned to a horizontal position and emptied before the next experiment.

Due to the flume’s dimensions, the region of interest (ROI) for image acquisition did not cover the entire flume length. Our experimental images captured a length of 44 cm focused on the flow’s leading edge, near the downhill end of the flume. Thus, our experimental results involved 42% of the whole bulk. Full image acquisition of the entire avalanche would have implied lower image resolution, and the benefits in terms of physical insights would have been limited because the recirculation of large particles in our experiments took place within the avalanche’s leading edge, whereas the unimaged flow behaved similarly to the flow in the ROI.

We were not able to capture the space immediately downstream of the front because the POM pieces appeared to move vertically to the camera, owing to the perspective and camera position. These pieces reflected an important amount of the light emitted by the laser, so the inclusion of the downstream end would have altered the light intensity and image contrast of the images and thereby would have impeded particle identification.

### Belt velocity

We can consider $u_{b}$ as an input parameter or a measurement. Indeed, although $u_{b}$ was set before experimental image acquisition, its value varied with $\theta$, the load on the belt and the friction imposed by the belt’s pieces. Many processes can alter motor torque and thereby belt velocity (see §4). Instead of calibrating of $u_{b}$ as a function of the electrical power supplied to the belt engine—a process fraught with uncertainties—we decided to measure its velocity directly after the desired stationary regime was achieved. Table 2 recaps the features of this work’s experimental dataset, with the measured $u_{b}$ values shown in the last column.

### Role of the interstitial fluid

We evaluated the influence of the interstitial fluid in our experiments via the calculation of non-dimensional numbers. To address how an interstitial fluid could lubrify particle contacts, Ancey et al. (1999b) determined the ratio between the weight and lubrication forces acting on grains. This ratio yielded the non-dimensional number

21 $$\begin{aligned} N=\frac{6\pi }{\Phi \varepsilon }\frac{\eta {\dot{\gamma }}}{\Delta \varrho gh}, \end{aligned}$$

where $\varepsilon \approx 0.05$ defines the lubrication layer thickness ($\varepsilon d$) and $\Delta \varrho =\varrho =\varrho _{*}-\varrho _{*}^{f}$. Using our experimental parameters, we obtained $N=5\times 10^{-4}\ll 1$. This value meant that in our experiments, contacts between particles were not lubricated by the interstitial fluid.

For granular avalanches immersed in an ambient fluid, Courrech du Pont et al. (2003) determined three regimes depending on the Stokes number

22 $$\begin{aligned} \mathrm {St}=\frac{1}{18\sqrt{2}}\frac{\sqrt{\varrho_{*}\Delta\varrho gd \mathrm {sin}\, \theta }}{\eta }d, \end{aligned}$$

and a grain-fluid density ratio $r=(\varrho _{*}/\varrho _{*}^{f})^{1/2}$. The particle Reynolds number can be obtained from the ratio of St and $r$, i.e. $\hbox {Re}_{p}=\mathrm {St}/r=({\varrho^{f}_{*}\Delta\varrho } gd \mathrm {sin}\, \theta )^{1/2}d/(18\sqrt{2}\eta )$ Using our experimental parameters, we found that the St number ranged from 16.35 to 37.32 with a $r=$ 1.53, which situated our experiments within the inertial regime—see Fig. 3 in Courrech du Pont et al. (2003). Within this regime, the dynamics of granular avalanches depend on the interstitial fluid principally via buoyancy effects. Alternatively, Ancey et al. (1999a) showed that the behaviour of granular suspensions could be framed with only three numbers; the aforementioned St number, *I* (Eq. 5) and the Leighton number defined as

23 $$\begin{aligned} \mathrm {Le}=\frac{\eta d {\dot{\gamma }}}{2\varepsilon \sigma _{z}}. \end{aligned}$$

Both *I* and Le numbers were very small in our experimental avalanches, with values around $1.8\times 10^{-2}$ and $2\times 10^{-6}$, respectively (for both, monodisperse and bidisperse experiments). According to Ancey et al. (1999a), these values placed our experiments in the frictional regime (Le$\ll 1$ and $I\ll 1$), with a minor role played by the interstitial fluid and particle collisions.

## Results

### Monodisperse experiments

**Fig. 4:**
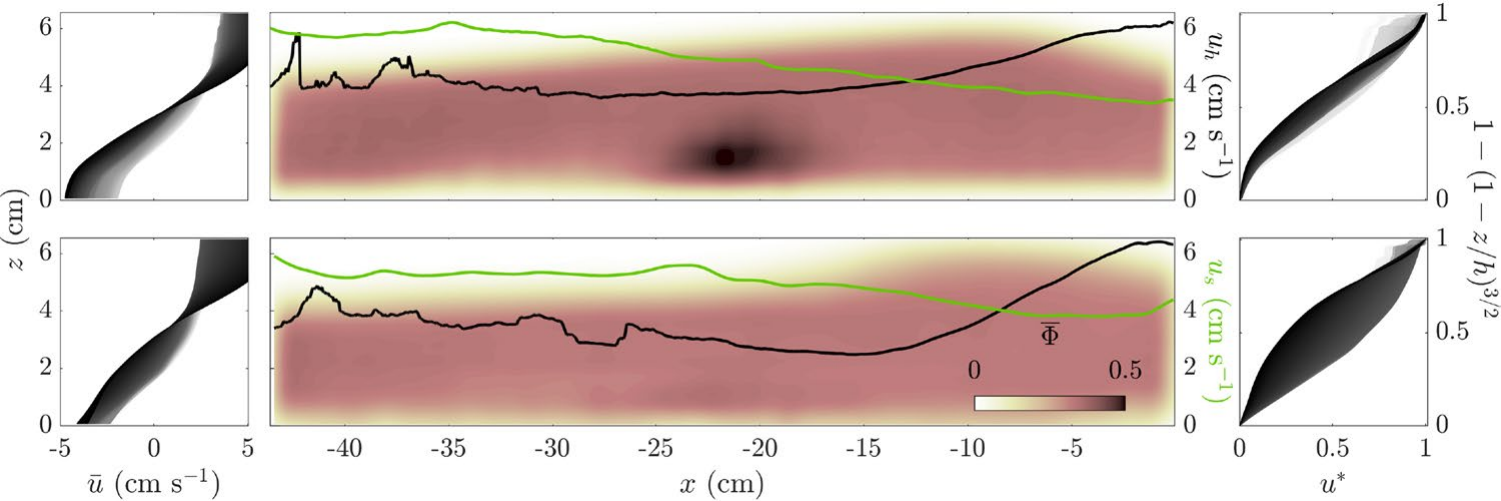
Time-averaged velocity profiles ${\bar{u}}={\bar{u}}(z)$ (left column); time-averaged bulk’s solids volume fraction field ${\overline{\Phi }}$, and free-surface velocity $u_{h}$ (*black* line) and slip $u_{s}$ (*green* line) velocity (centre column); and normalised velocity profiles $u^{*}=u^{*}(1-(1-z/h)^{3/2})$ (right column). Top and bottom rows show results for monodisperse 6-mm and 8-mm experiments (Experiments 1 and 6 in Table 2, respectively). The velocity profiles are plotted in grayscale from white to black, from the back to the front of the flow’s ROI, respectively

The monodisperse avalanches showed two distinctive flow sections: one at the front and the other at the back, with a marked transition between the two. Towards the flow front, at the flume’s downhill end, a convective, bulged region was observed. This front bulge was similar to that studied recently by Denissen et al. (2019) for a bidisperse flow. Henceafter, we refer to this region as the flow’s convective-bulged front, or expanded front. In the rest of the conveyed volume, towards the flume’s uphill end, the particle flow transitioned into a well-arranged structure of particle layers that moved on top of each other. Naturally, this compact, ordered region was referred to as the layered flow. To illustrate these flow regions, Fig. 3 shows an experimental image and its corresponding extracted fields. This image was taken from Experiment 1 at $t=$ 229.25 s, and its bulk volumetric concentration $\Phi$, longitudinal (in the direction of the flow) velocity *u* and vertical velocity *w* fields are plotted on top of it. Figure 3 reveals the particle structures in the background and a glimpse of the described flow regions. Overall bulk concentrations $\Phi$ varied between the described regions. The front showed a more diluted flow $\Phi \approx 0.3$, whereas the tail was more concentrated ($\Phi \approx 0.5$). The flow height $h$ also showed marked differences, with height at the front reaching its maximum at $h=$ 6 cm, or $\approx 10d_{s}$, whereas it was close to 4 cm at the back, or $\approx 7d_{s}$.

To highlight some sections of the velocity field, we plotted profiles along the inclined flume’s longitudinal and transverse sections (see continuous white plots in Fig. 3a and b). The velocity field measurements revealed a quasi-uniform behaviour for *u*(*z*), with particles at the top moving faster than those at the bottom and a significant basal-slip condition, which was to be expected in such granular experiments (Louge and Keast 2001; Ancey 2001; Hsu et al. 2008). Vertical velocity $w(x)$ profiles were notably less consistent along the flow, where we observed much more vertical particle movement in the flow’s leading edge than in its tail, where particle layers just moved on top of each other. As mentioned, the flow could be separated into two sections at $x\approx -25$ cm, measured longitudinally from the flume’s downhill end. A sudden change of $w\approx 4$cm $\hbox {s}^{-1}$ marked the transition between the tail and leading-edge sections, from where particles started to recirculate within the bulged front. Only particles that had become tightly attached to the belt managed to escape the leading edge and, after reaching the flume’s uphill end, they were reincorporated into the avalanche.

Time-averaged ${\bar{\Phi }}$, ${\bar{u}}$ and ${\bar{w}}$ fields were calculated to refine these general observations. We measured the time-averaging velocity profiles $u(z)$ to show that velocity scaled with $h^{3/2}$. When time-averaged, velocity fields became smoother and the ${\bar{u}}(z)$ profiles showed consistent behaviour in the longitudinal direction (Fig. 4 left column). The velocity profiles ${\bar{u}}_{z}$ are plotted in Fig. 4 as increasingly dark lines (grayscale) as we took vertical velocity profiles from the upstream (in white) to the downstream end (in black) of flow’s ROI.

In general, these ${\bar{u}}(z)$ profiles showed Bagnold-like characteristics, but subject to a strong basal slip $u_{s}$ (Fig. 4 right column). Figure 4 shows a normalised velocity profile $u^{*}$ defined as

24 $$\begin{aligned} u^{*}=\frac{{\bar{u}}-u_{0}}{u_{h}-u_{0}} \end{aligned}$$

where $u_{h}={\bar{u}}(h)$ is the surface particle velocity, and $u_{0}={\bar{u}}(0)$ is the basal particle velocity, which in turn defined the slip velocity as the difference between belt speed and basal velocity, i.e. $u_{s}=u_{b}-|u_{0}|$. Therefore, $u^{*}$ is the time-averaged velocity field $\bar{u}$ normalised to the velocity difference between the base and the surface, and it is plotted as a function of $1-(1-z/h)^{3/2}$ (Fig. 4). We considered this expression for normalised velocity to correct the influence of basal slip $u_{s}$ on the overall velocity profile, which was negligible close to $z=h$. Close to the flow’s free-surface, most $u^{*}$ profiles adjusted well to $1-(1-z/h)^{3/2}$, which is characteristic of a Bagnold-like profile (Bagnold 1954; Silbert et al. 2001). In terms of longitudinal variation in the $u^{*}$ profiles, we saw that, towards the front, slip decreased and surface velocities increased (see $u_{s}$ and $u_{h}$ in the centre-column subplots of Fig. 4). As a result, the $u^{*}$ profiles shown in Fig. 4’s right column subplots were in better agreement with the Bagnold scaling as we approached to the flow’s leading edge (darker lines are for vertical sections closer to the flume’s downhill end). However, at the very front of the flow, the agreement with the theoretical Bagnold profile decreased again. From a hydraulic point of view, this behaviour might be related to the changes in $u_{s}$, $u_{h}$, and the free-surface profile.

**Fig. 5:**
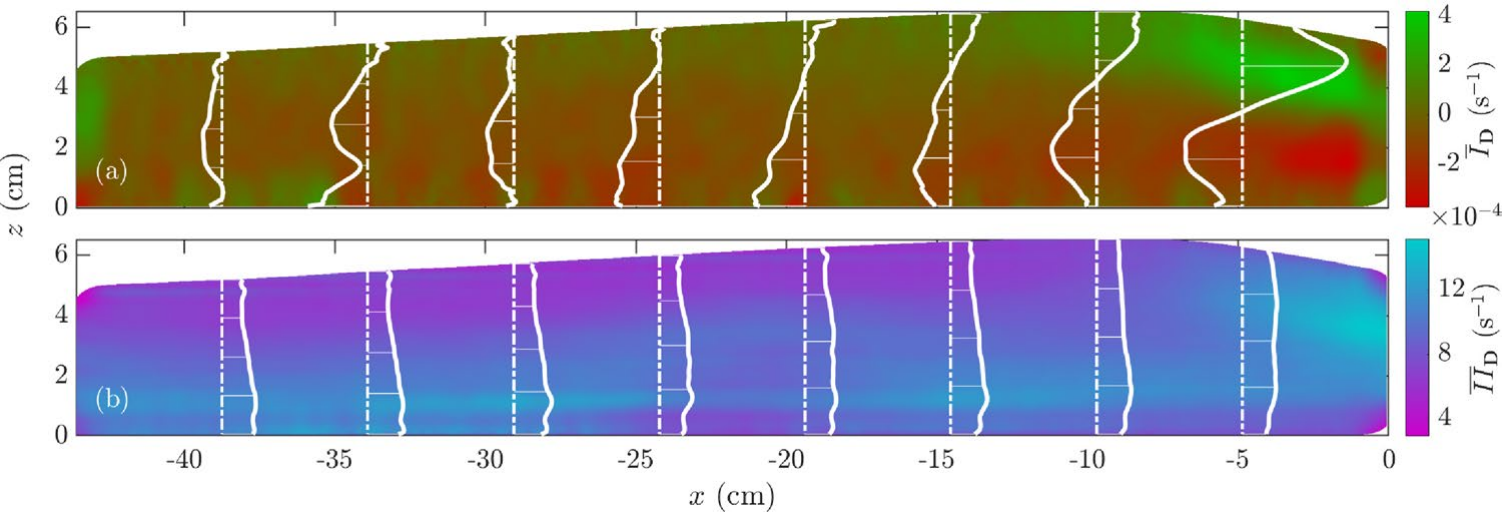
Time-averaged strain-rate tensor invariants in Experiment 1:** a** expansion rate ${\overline{I}}_{{\varvec{D}}}$ and** b** shear rate ${\overline{II}}_{{\varvec{D}}}$. The vertical dashed lines correspond to vertical profiles and the corresponding values for the strain-rate tensor invariants are plotted as continuous white lines

We determined the strain-rate tensor invariants to quantify how expanded or sheared the two flow regions were. Figure 5 shows the time-averaged expansion rate ${\overline{I}}_{{\varvec{D}}}$ and shear rate ${\overline{II}}_{{\varvec{D}}}$ fields corresponding to the strain-rate tensor’s time-averaged first and second invariants (see Sect. 2.3). Our results indicated that the leading edge was highly sheared and expanded, with a marked vertical gradient for ${\overline{I}}_{{\varvec{D}}}$ and constant ${\overline{II}}_{{\varvec{D}}}$ close to the front. To the rear, ${\overline{I}}_{{\varvec{D}}}$ fluctuated around 0, and ${\overline{II}}_{{\varvec{D}}}$ showed a negative gradient to the flow’s free-surface, with higher values at the bottom, as we would expect from the imposed boundary condition. Qualitatively, expansion and shear rates were significantly higher in regions where flow height was also high and ${\overline{\Phi }}$ was low, right to the convective front. These observations were also related to low $u_{s}$ values, as shown in Fig. 4. A decrease in basal slip might explain the avalanche’s blunt front appearance and high expansion rate, due to the more effective shear transmission from the belt to the bulk.

### Bidisperse experiments

**Fig. 6:**
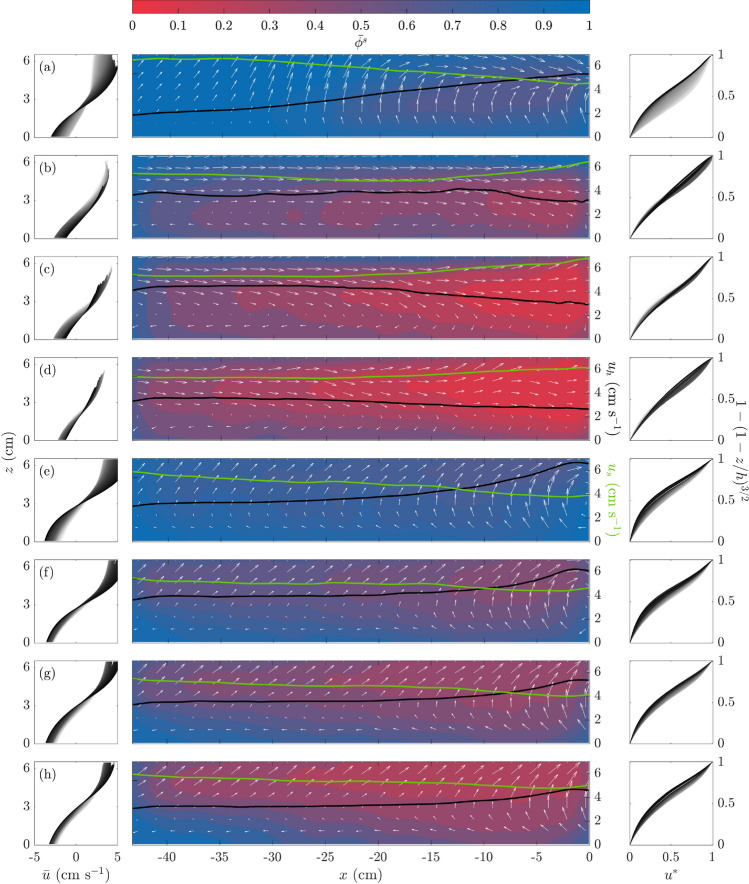
Time-averaged velocity profiles ${\bar{u}}={\bar{u}}(z)$ (left column); time-averaged small particle concentrations ${\bar{\phi }}^{s}$, free-surface velocities $u_{h}$ (*black* lines) and slip velocities $u_{s}$ (*green* lines) (centre column); and normalised velocity profiles $u^{*}=u^{*}(1-(1-z/h)^{3/2})$ (right column).** a**–**d** are for Experiments 2–5, and** e**–**h** are for Experiments 7–10 (see Table 2). White arrows represent the normalised velocity fields ${\bar{u}}/\max _{\forall x,z} ({\bar{u}})$ and ${\bar{w}}/\max _{\forall x,z} ({\bar{w}})$. Velocity profiles in the left and right columns are plotted in grey scale, from white to black, from the upstream end to the downstream end of the flume’s ROI

Adding large particles did not substantially change our avalanches’ overall dynamics. When the concentration of large particles was low ($\Phi ^{l}=$ 10–20%), the leading edge concentrated those large particles, recirculating them. For $\Phi ^{l}>20\%$, the well-defined regions observed at low concentrations became less apparent, whereas other structures emerged. Large particles were often dragged uphill, past the transition between the bulged-front and layered-tail regions, altering the characteristic structures observed in the monodisperse experiments. When we time-averaged the velocity and concentration fields, we still observed the convective leading edge. As a result of particle-size segregation, large particles were found predominantly at the free surface and within the leading edge, due to strong segregation fluxes in the middle of the bulk. This behaviour probably reflected a more effective transmission of shear close to the front, or low slip, which was observed in the monodisperse experiments.

**Fig. 7:**
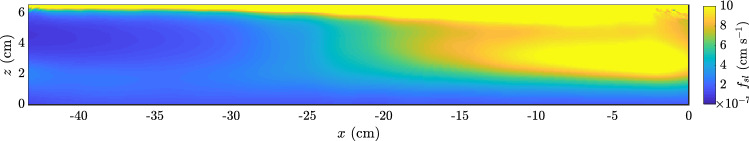
Segregation flux $f_{sl}$ for the $\Phi ^{s}=$ 90 % experiment (Experiment 2, Tab. 2). $f_{sl}$ was calculated using the formulation suggested by Trewhela et al. (2021a) and simplified in Eq. 13

Figure 6 shows the time-averaged small particle concentration field $\bar{\phi ^{s}}$ for our experiments (Fig. 6, centre column). As expected from size segregation theory, the time-averaged concentration fields show an inversely-graded bulk towards the flume’s downhill end. We can infer from Fig. 6 that in experiments with larger *R* values (Experiments 2–5, (a)–(d) in Fig. 6), large particles recirculated within the flow’s leading edge. This large particle concentration resulted from a relatively faster segregation flux $f_{sl}$ for larger $R$ values. To support this interpretation, we determined the segregation flux $f_{sl}$ using the empirical expression 13 (Fig. 7). For this calculation, we assumed a hydrostatic pressure distribution, and the $\phi ^{s}$ and ${\dot{\gamma }}=2 {\overline{II}}_{{\varvec{D}}}$ fields were determined from coarse-grained experimental data. Results presented in Fig. 7 indicate that the segregation flux was highest in the convective-front region. This was closely connected with the shear-rate distribution shown in Fig. 5 for the monodisperse case, since $f_{sl}\sim {\dot{\gamma }}$. The high values for $f_{sl}$ at the front were still smaller than the $w$ values at the transition between flow regions, presented in Fig. 3 ($w\approx 4$ cm $\hbox {s}^{-1}$).

Segregation-induced large-particle recirculation was observed in all the experiments and is shown via normalised velocities, ${\bar{u}}/\max _{\forall x,z} ({\bar{u}})$ and ${\bar{w}}/\max _{\forall x,z} ({\bar{w}})$, in the form of white arrows (Fig. 6, centre column). For $R>2$, expansion rates were found to be related to more efficient, and hence faster, segregation rates (Trewhela et al. 2021b), which was evidenced by our large particles in Experiments 2–5. Our results for $d_{s}=$ 6 mm, i.e. $R=$ 2.33, showed that large particles are probably to be constrained to the bulged-front, seen in Fig. 6a–d. For the experiments with $R=$ 1.75 in Fig. 6 (e)–(h), we observed a less marked segregated state at the front with lower large-particle concentrations, a sign that large particles were more homogeneously distributed along the flume.

Breaking size segregation waves were observed in all the bidisperse avalanches. We can infer from Fig. 6 that the breaking-size segregation wave structure was similar to that observed by van der Vaart et al. (2018). From near the surface of the downstream end of the flow, large particles (red colour intensities in Fig. 6) fell onto the very front of the avalanche where they were overrun by the flow and dragged back into the bulk. Eventually, these large particles segregated and rose back to the surface onto the front, recirculating. The lens-shaped region (Gray and Ancey 2009; Johnson et al. 2012; van der Vaart et al. 2018) where large and small particles were interchanged as a result of shear-induced segregation, can be seen in the middle part of the avalanche, between $-40<x<-10$ cm for (a) to (h) in Fig. 6. Concentration gradients of ${\bar{\phi }}^{s}$ in this region indicate an apparent mixing, where large particles rise and small particles percolate as a result of segregation. Our results also showed that changes in the overall particle concentrations between different experiments induced variations in the characteristics of the breaking-size-segregation waves. As we increased the overall concentration of large particles, they formed a thicker layer within the leading edge and the lens region extended uphill of the flow, disrupting the layered region described in Sect. 6.1.

The results shown in Fig. 6 follow the trend observed in the monodisperse media experiments. Slip $u_{s}$ was lower, with values that changed along the direction of the flow and ranged from 40% to 80% of $u_{b}$ (Fig. 6, green lines in centre column). From Fig. 6, we infer that the addition of large particles regularised slip by making the longitudinal gradient less steep than the low $\Phi ^{l}$ and monodisperse media experiments. Surprisingly, the experiments with $d_{s}=$ 6 mm showed an inversion of the $u_{s}$ profile when $\Phi ^{l}$ was increased: a higher $u_{s}$ was measured near the flow when $\Phi ^{l}>10\%$. However, this result was not consistent with the experiment made using $d_{s}=$ 8 mm, which suggests that bed roughness played an important part in shear transmission and should be considered when analysing the slip effect.

A natural question following the addition of large particles was whether the velocity profiles were Bagnold-like or not. In similar fashion to Fig. 4, we plotted the time-averaged velocity profiles ${\bar{u}}_{z}$ as we moved longitudinally from the upstream end (in white) to the downstream end (in black) of the flow’s ROI. Fig. 6’s right column shows that $u^{*}$ came close to $1-(1-z/h)^{3/2}$, indicating a Bagnold-like velocity profile (Bagnold 1954; Mitarai and Nakanishi 2005) for the bidisperse avalanches. Even though this result might have been expected, we found that velocity profiles were consistently uniform. Similar to what was observed for monodisperse materials, basal slip tended to skew the profiles, particularly close to the belt, but further from the bottom, profiles were in good agreement with the Bagnold scaling. Average particle concentration influenced results in terms of consistency, as discussed: when $\Phi ^{l}$ was increased, the slip all along the base became less variable and the velocity profiles matched the function $1-(1-z/h)^{3/2}$.

**Fig. 8:**
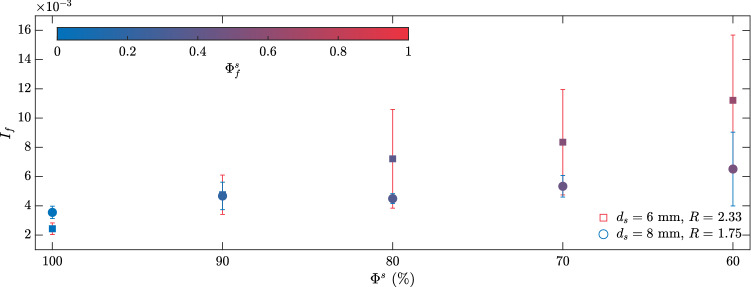
Averaged inertial number $I_{f}$ as a function of $\Phi ^{s}$ for the monodisperse and bidisperse experiments. The colour on the markers indicates the average small particle local concentration for the entire flow front $\Phi ^{s}_{f}=(d_{f}-d_{l})/(d_{s}-d_{l})$ (see Eq. 17). Error bars in colours (red and blue for $d_{s}=$ 6 and 8 mm, respectively) plot the standard deviation of the measured *I* values

Observing Bagnold-type velocity profiles, we estimated an inertial number $I$ using Eq. 7 and the experimental parameters for each profile

25 $$\begin{aligned} I=\frac{3Kd_{H}}{2\sqrt{g\mathrm {cos}\,\theta h^{3}}}, \end{aligned}$$

where *K* is the empirical parameter such that ${\bar{u}}-u_{0}=K[1-(1-z/h)^{3/2}]$ (determination coefficients $r^{2}$ were larger than 0.85 for all velocity profiles) and $d_{H}=1/h\int ^{h}_{0} d \mathrm {d}z$ is the depth-average of $d$. The $I$ and $d_{H}$ calculations were averaged for the whole avalanche front to yield an inertial number for each experiment $I_{f}$, which are shown in Fig. 8 as a function of $\Phi ^{s}$.

The inertial number $I_{f}$ increased as large particles were added to the bulk, which was to be expected (see Eq. 5, i.e. $I\sim d$). The standard deviation of $I_{f}$ also increased with $\Phi ^{s}$ due to changes in local particle concentrations, which were reflected in a variable longitudinal distribution of $d_{H}$. The monodisperse experiments showed a larger value of $I_{f}$ for $d_{s}=$ 8 mm, and in general they exhibit small deviations from their mean values. Unexpectedly, $I_{f}$ values for bidisperse experiments with $d_{s}=$ 6 mm were consistently larger than those for $d_{s}=$ 8 mm despite the higher *d* values. These results can be explained by segregation and large particle distribution along the leading edge. As shown in Fig. 6, large particle concentration at the leading edge was markedly higher in Experiments 2-5 than in Experiments 7-10 due to a larger $R=$ 2.33 value, which comparatively to $R=$ 1.75 should present faster segregation rates (Trewhela et al. 2021a, b). These higher large particle concentrations at the front in Experiments 7-10 result in larger $d_{H}$ values, hence larger $I_{f}$ values. This increase in $I_{f}$ due to large particle recirculation comes with sharper differences between $d_{H}$ values at the front and back of the flow, which explained the larger deviations as we increased $\Phi _{s}$. To visualize the changes in the flow’s concentration-averaged diameter, we took the mean value of the $d_{H}$ for the entire front to obtain $d_{f}$. The obtained values were within the $[d_{s},d_{l}]$ range, so to make them comparable, we computed the relative diameter value $(d_{f}-d_{l})/(d_{s}-d_{l})$ which in virtue of Eq. 17 corresponded to the average small particle concentration for the entire flow $\Phi ^{s}_{f}$. Unsurprisingly, $\Phi _{f}$ increased with $\Phi ^{s}$ but were not equal, due to our focus on the flow’s leading edge.

## Conclusions

Using a specially constructed inclined conveyor belt, we ran experiments to study granular avalanches. All ten experiments, carried out using monodisperse or bidisperse media, exhibited a quasi-uniform steady behaviour characterised by a convective front at the downhill end of the inclined flume and a particle-layered tail towards the flume’s uphill end. Our experimental results revealed characteristics and structures typical of granular flows, which have been described in the literature. These features included blunt fronts (Denissen et al. 2019), breaking size-segregation waves (Thornton and Gray 2008; Gray and Ancey 2009; Johnson et al. 2012; van der Vaart et al. 2018) and crystallisation (Tsai and Gollub 2004).

Even for bidisperse media, time-averaged velocity profiles showed a $h^{3/2}$ scaling consistent with Bagnold’s rheology and in agreement with earlier observations (Silbert et al. 2001; Mitarai and Nakanishi 2005), and the $\mu (I)$ rheology (Jop et al. 2006). In this respect, the consistent behaviour exhibited by the $u^{*}$ profiles suggests the existence of an equivalent particle diameter dependent on $d_{\nu }$ and $\phi ^{\nu }$, as defined by Eq. (15) (Tripathi and Khakhar 2011). This finding prompted the calculation of the flow’s inertial number $I$ as a function of the depth and concentration-averaged diameter. We found that the inertial number increased when large particles concentration were higher, but that brought along larger deviations from the flow’s mean *I* value. These deviations were a result of sharp and segregated particle distributions along the main direction of the flow.

Finally, these velocity profiles could be used as inputs to models coupling size segregation and granular avalanches as suggested by Gray and Ancey (2009). We moved a step further in that direction by computing the segregation flux $f_{sl}$ using the expression proposed by Trewhela et al. (2021a). We found that $f_{sl}$ was high within the avalanche’s leading edge, and this was further confirmed by large-particles recirculation and high values for both strain-rate tensor invariants (Trewhela et al. 2021b).

Various velocity and slope conditions should be explored to identify similarities and differences with the results presented here. The influence of the belt’s roughness and the vertical boundaries created by the upper rollers could also be addressed, but eliminating their influence on the flow is currently impractical and outside this article’s scope. We believe that further work in that direction would not change the significance of the present experiments. Nonetheless, this experimental conveyor belt setup proved to be useful for the visualisation and study of the internal dynamics of granular flows.

## Figures and Tables

**Fig. 9:**
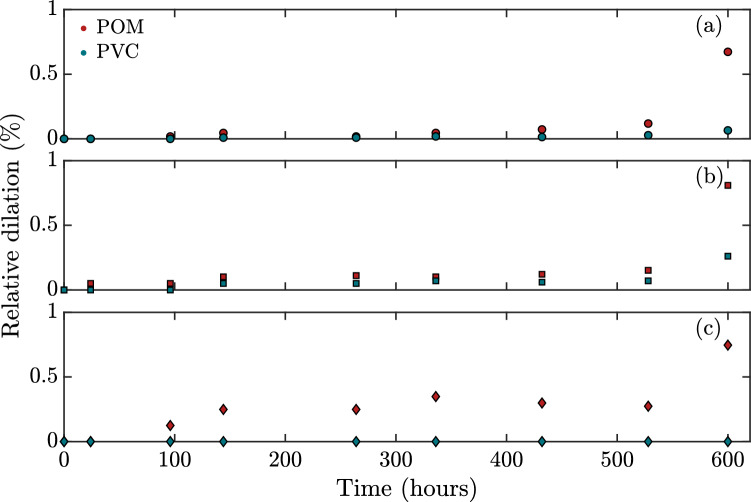
Measurements of relative dilation at different fluid-exposure times for identical POM (red) and PVC (turquoise) belt pieces. Dimensions $L^{i}$ correspond to $i:=$ 1 for length,** a**; 2 for width,** b**; and 3 for height** c**. No stresses were applied to the pieces

## Appendix: Setup-related difficulties

### Plastic-fluid interactions

Two important objectives of this setup were minimising the fluid volume used and the investigators’ exposure to harmful, flammable vapours; we therefore devised an enclosed flume. Achieving these objectives required sealing the setup to avoid leaks and spillages, which can be extremely dangerous when dealing with flammable fluids. Although this might seem simple at the outset, using non-conventional fluids led to interactions with the setup’s components. Sealing had to be done with plastics, but many of them reacted chemically with the ethanol and benzyl-alcohol mixture. For example, acrylics like poly (methyl methacrylate) (PMMA) were rapidly dissolved, and most rubbers lost some of their elastic properties—a feature fundamental for sealing. These materials had to be scrapped after a couple of hours or days of exposure to the fluid.

Among plastics, polyoxymethylene (POM) and polyvinyl chloride (PVC) resisted long exposure to the fluid well. We observed no important changes in their material properties. However, and in general, these plastics are slightly porous and tend to absorb small amounts of fluid when immersed for a long time. Even though that absorption was very small, we noticed that after long use, friction between the belt pieces and the flume’s grooves increased to a point where the motor jammed. Not only did friction increase, but compression between the POM pieces became notably higher, which desynchronised them from the geared wheel. To determine how large the fluid absorption was, we measured the changes in length of two identical pieces of differing materials.

Figure 9 shows measurements of two immersed belt pieces: one made of POM and one of PVC. Measurements were made after different fluid-exposure times, of up to 600 hours of submergence. To quantify the expansion, the material’s relative dilation was calculated as

26 $$\begin{aligned} \text {Relative\;dilation}\;(\%)=\frac{|L^{i}-L^{i}_{0}|}{L^{i}_{0}}, \end{aligned}$$

where $L^{i}(t)$ is the post-submergence measurement in mm ($i={1,2,3}$ for length, width and height, respectively) and $L^{i}_{0}$ is the pre-submergence measurement in mm. We observed that POM pieces swelled much more, and more steadily, than their PVC counterparts. This swelling was sufficient to explain the problems created in the experimental setup. An expansion of $\sim$1% in belt depth or length may seem negligible, but with 300 adjacent pieces, this expansion was equivalent to adding three extra pieces to the belt system. More than one extra piece was enough to desynchronise the geared wheel, which ended up blocked by the bolts getting caught in its teeth. Regarding side friction, this increased consistently with the extra compression resulting from the pieces expanding in the already compressed belt system. As we observed after our experiments, friction became great enough to gradually bring the motor to a halt.

In general, PVC expanded by less than half of POM’s expansion, encouraging us to use PVC in future experiments. PVC is also opaquer than POM, and thus laser reflection close to the belt was lower and image definition was improved.

Rubbers and silicones were required to seal setup joints and the screwed pieces that needed to be routinely changed between experiments. When exposed to the RIM fluid, rubber rapidly loses elasticity, and after long exposure it partially dissolves, leading to leaks and fluid pollution, both of which are detrimental to safety and finances. We tested different types of rubber and silicone with little-to-no success. To the best of our knowledge, only Viton, a fluoroelastomer, was adequate for the o-rings or other rubber pieces. Common silicone could not seal the setup and was rapidly dissolved. Silicone LOCTITE SI 5910 managed to endure exposure to the fluid effectively for long periods without leakage.

### Pollutants and filtering system

The conveyor belt pieces inevitably produced friction between themselves, the grooves and the rollers. Most of the friction was against aluminium and the scraping released a very fine black dust that spreads throughout the setup. Long-running experiments accumulated enough dust to reduce fluid transparency, obstructing the laser and reducing image quality. To enhance image definition and increase light intensity, we changed acquisition parameters, like the gain and/or reduced the lens’ focal ratio. Nevertheless, the black dust concentration would increase to a point where these improvements became pointless.

A filtering system was devised to remove the dust from the fluid. We could have used a wet sieving/filtering system, such as a Retsch AS200, but knowing nothing about its components and not wanting to damage it with exposure to our fluid, we decided to try a simpler method. A large reservoir dripped fluid into a funnel containing 40 $\mu\text{m}$ filter paper. We were able to filter the fluid entirely in approximately one day, which was a reasonable amount of time in which to prepare the next experiment. With twice the amount of fluid, we were able to rotate the fluid batches and keep the amount of dust under control, thus not harming image quality. A reduction in dust production, via improvements such as stainless steel grooves, could certainly enhance the setup’s performance and image quality in the future, but these were not used for this work.
